# Osteoarthritis-patterns, cardio-metabolic risk factors and risk of all-cause mortality: 20 years follow-up in patients after hip or knee replacement

**DOI:** 10.1038/s41598-018-23573-2

**Published:** 2018-03-27

**Authors:** G. Büchele, K. P. Günther, H. Brenner, W. Puhl, T. Stürmer, D. Rothenbacher, R. E. Brenner

**Affiliations:** 1 Institute of Epidemiology and Medical Biometry, Ulm University, Helmholtzstraße 22, 89081 Ulm, Germany; 2 University Center of Orthopaedics and Traumatology, University Medicine Carl Gustav Carus Dresden, TU Dresden, Dresden, Germany; 3 Division of Clinical Epidemiology & Aging Research, German Cancer Research Center (DKFZ), Heidelberg, Germany; 4 Network Aging Research, University of Heidelberg, Heidelberg, Germany; 5 Department of Orthopedics(emeritus), University of Ulm, Ulm, 89081 Germany; 6 Department of Epidemiology, Gillings School of Global Public Health, University of North Carolina at Chapel Hill, Chapel Hill, NC USA; 7 Department of Orthopedics, Division for Biochemistry of Joint and Connective Tissue Diseases, University of Ulm, Ulm, 89081 Germany

## Abstract

Osteoarthritis (OA) is a common musculoskeletal disorder and occur in different patterns. However, its impact on long-term all-cause-mortality is inconclusive. Study aims: Investigate 20-year all-cause-mortality in patients with hip/knee arthroplasty (recruited 1995/1996, N = 809) from the Ulm Osteoarthritis Study-cohort, in comparison to general population. Furthermore, to enlighten the triangle between baseline life-style and cardio-metabolic risk factors, phenotypic OA-patterns (laterality, generalization, cause) and all-cause-mortality. Mortality was assessed during 20 years follow-up. Standardized mortality ratios (SMR), adjusted odds ratios and hazard ratios (aHR) were calculated. After five years cohort-mortality was reduced compared to the general population, however 20 years later assimilated (SMR = 1.11; 95%-CI 0.73-1.49). OA-patterns were associated with age, cholesterol, and overweight/obesity. In comparison to primary OA decreased mortality was observed for patients with secondary OA (aHR = 0.76; 95%-CI 0.61-0.95) adjusted for age, smoking, overweight/obesity, diabetes, hypertension, cardiac insufficiency, uric acid, and lower cholesterol. There was no increased mortality in patients after 20 years follow-up compared to general population. Significantly decreased mortality in secondary compared to primary OA suggests a subtype-specific involvement of systemic co-factors in determination of all-cause-mortality. Because cardio-metabolic risk factors were associated with increased risk of bilateral OA and lower long-term survival, those risk factors should be consequently targeted in OA-patients.

## Introduction

Osteoarthritis (OA) is a very common musculoskeletal disorder. Prevalence of OA increases with age and is associated with functional disability and pain in the joints^1^. Furthermore, OA is a heterogeneous disease which may involve single or multiple joints. Beside the spine and hands, hip and knee are the most affected locations. Arthroplasty is an established treatment in symptomatic patients in order to restore functionality, relieve pain, and increase overall quality of life.

Epidemiologic studies have revealed that the importance of specific risk factors varies in OA subtypes. Besides genetics, age, sex, joint injury, and obesity; different systemic etiological factors such as inflammation, inflamed adipose tissue, and dyslipidemia are recognized risk factors for OA^2–6^. In addition, the presence of metabolic syndrome has been shown to determine the overall risk for development and progression of OA. In particular, the relationship of obesity and metabolic syndrome with OA has become increasingly evident^7^. Furthermore, different patterns of osteoarthritis (i.e. generalization, laterality, secondary cause) have been shown to be related to aspects of body composition, lipid levels, the metabolic syndrome, and other cardio-metabolic risk factors which may also point to shared pathophysiologic pathways^4,5^.

There is controversy with respect to the potential association between OA and an elevated risk of all-cause mortality^8^. Previous studies concerning all-cause-mortality in OA speculated about an increased risk in certain subgroups mainly due to cardiovascular disease and obesity related risk factors^7,9^. Therefore, understanding the relationship between cardio-metabolic phenotypes and obesity with the risk of various types of OA and the association with long-term mortality can help to further enlighten the complex relationship and to identify potential common pathophysiological pathways. This knowledge then might be used for identification of preventive as well as therapeutic approaches.

The aim of this investigation was to analyze 18- to 20-year all-cause-mortality in patients with hip or knee replacement from the Ulm Osteoarthritis Study cohort and to compare it with the mortality of the general population. Moreover, the association of multiple factors with patterns of OA and the relationship of baseline life-style as well as other factors such as BMI, serum lipids and cardiac comorbidities, low-grade systemic inflammation and phenotypic patterns of OA with all-cause mortality was analyzed.

## Results

**Figure 1:**
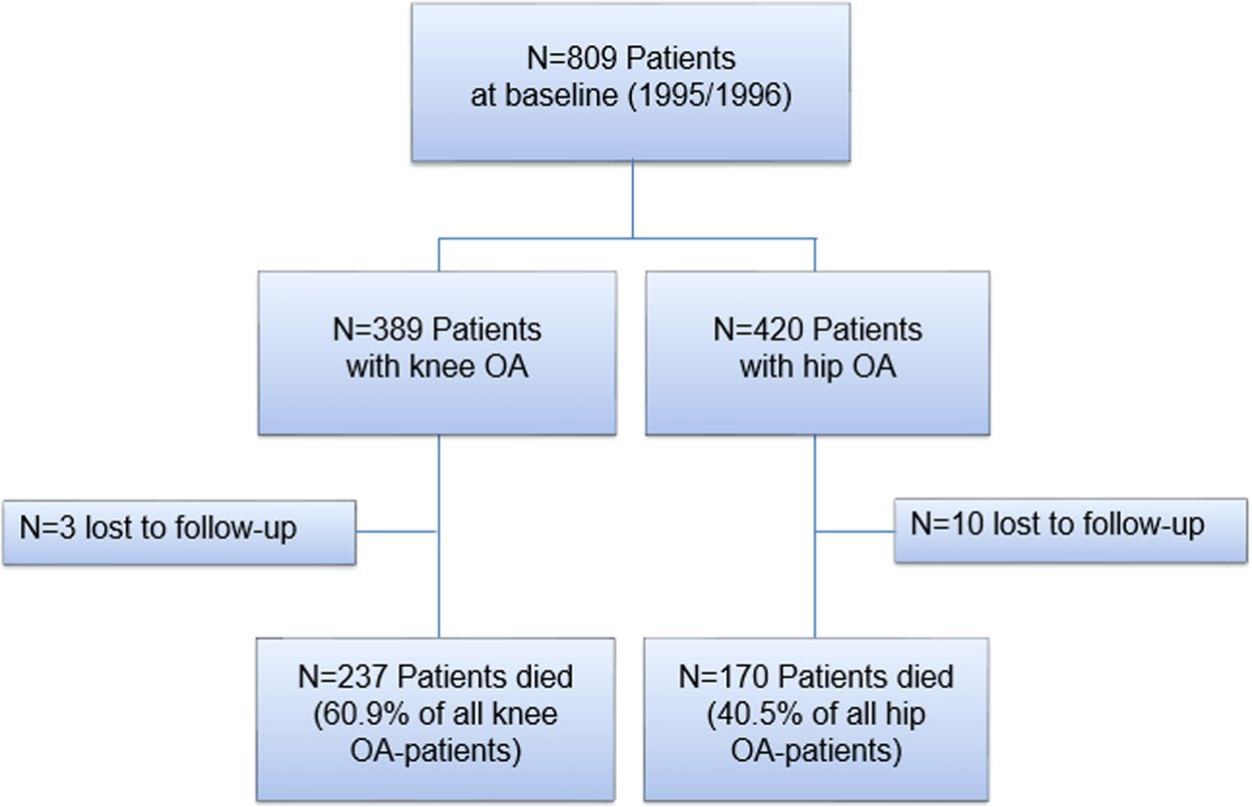
Patients flow chart and mortality during 20 years of follow-up. OA osteoarthritis, N number of patients.

In the baseline investigation there were N = 809 patients with knee (N = 389) or hip (N = 420) OA included (Fig. 1). At the end of the 20 year follow-up, N = 407 (50.3%) patients were deceased and n = 13 (1.6%) patients had been lost to follow-up. Median observation time was 18.4 years (Table 1). Patients with knee and hip OA differed with respect to proportion of female patients, smoking status, and self-reported history of overweight/obesity. Furthermore, median age of patients with knee OA was five years older and OA patients also exhibited more comorbidities. Bilateral OA was a common diagnosis among recruited patients (77% of all patients, Table 1). However, a generalized OA (defined by additional presence of multi-site hand OA) was manifest only in 21% of the participants. Secondary reasons for OA were documented in about one third (37%) of the patients. Knee OA was attended with a higher percentage of generalized OA and primary OA.

**Table 1:** Patient characteristics at baseline.

(N, % of total cohort)	Total		Hip		Knee	
Total cohort	809	(100.0%)	420	(51.92%)	389	48.08%)
Sex female	504	(62.3%)	221	(52.6%)	283	(72.8%)
Age (years; N = 809; Median, Q1-Q3)	65	(58–70)	62	(55–68)	67	(63–71)
25–<60 y	236	(29.2%)	182	(43.3%)	54	(13.9%)
60–<70 y	351	(43.4%)	153	(36.4%)	198	(50.9%)
70–75 y	222	(27.4%)	85	(20.2%)	137	(35.2%)
Smoking: Former smoker	238	(29.4%)	141	(33.6%)	97	(24.9%)
Current smoker	102	(12.6%)	70	(16.7%)	32	(8.2%)
BMI (kg/m²; N = 809; Median, Q1-Q3)	27.9	(25.5–30.9)	26.8	(24.5–29.5)	29.2	(26.5–32.1)
<25 kg/m²	181	(22.4%)	126	(30.0%)	55	(14.1%)
25–<30 kg/m²	375	(46.4%)	200	(47.6%)	175	(45.0%)
30–<35 kg/m²	200	(24.7%)	76	(18.1%)	124	(31.9%)
>=35 kg/m²	53	(6.6%)	18	(4.3%)	35	(9.0%)
History of overweight/obesity	466	(57.6%)	191	(45.5%)	275	(70.7%)
Diabetes mellitus type 2	70	(8.7%)	28	(6.7%)	42	(10.8%)
Gout	100	(12.4%)	43	(10.2%)	57	(14.7%)
Hypertension	415	(51.3%)	190	(45.2%)	225	(57.8%)
Cardiac infarction	34	(4.2%)	18	(4.3%)	16	(4.1%)
Cardiac insufficiency	153	(18.9%)	57	(13.6%)	96	(24.7%)
Hypercholesterolemia	259	(32.0%)	127	(30.2%)	132	(33.9%)
Unknown	123	(15.2%)	64	(15.2%)	59	(15.2%)
Cholesterol (mmol/l; N = 683; Median, Q1-Q3)	5.7	(5.1–6.4)	5.6	(5.0–6.3)	5.8	(5.2–6.4)
Uric acid (mmol/l; N = 699; Median, Q1-Q3)	315.4	(265.0–376.0)	315.4	(267.8–376.0)	313.5	(257.5–375.5)
hs-CRP (mg/l; N = 770; Median, Q1-Q3)	2.52	(1.22–4.97)	2.59	(1.19–4.96)	2.47	(1.26–5.00)
Bilateral OA	622	(76.9%)	330	(78.6%)	292	(75.1%)
Unilateral OA	114	(14.1%)	72	(17.1%)	42	(10.8%)
Unknown	73	(9.0%)	18	(4.3%)	55	(14.1%)
Generalized OA	171	(21.1%)	64	(15.2%)	107	(27.5%)
Not-generalized OA	468	(57.8%)	268	(63.8%)	200	(51.4%)
Unknown	170	(21.0%)	88	(21.0%)	82	(21.1%)
Secondary OA	296	(36.6%)	170	(40.5%)	126	(32.4%)
Primary OA	489	(60.4%)	238	(56.7%)	251	(64.5%)
Unknown	24	(3.0%)	12	(2.9%)	12	(3.1%)
**20 years follow-up**						
Deceased (N,%)	407	(50.3%)	170	(40.5%)	237	(60.9%)
Observation time (years; Median, Q1-Q3)	18.4	(11.4–19.2)	18.6	(12.3–19.3)	16.7	(11.1–19.1)

N number of patients; Q1 first quartile; Q3 third quartile.

### Mortality of patients with OA compared to general population

**Figure 2:**
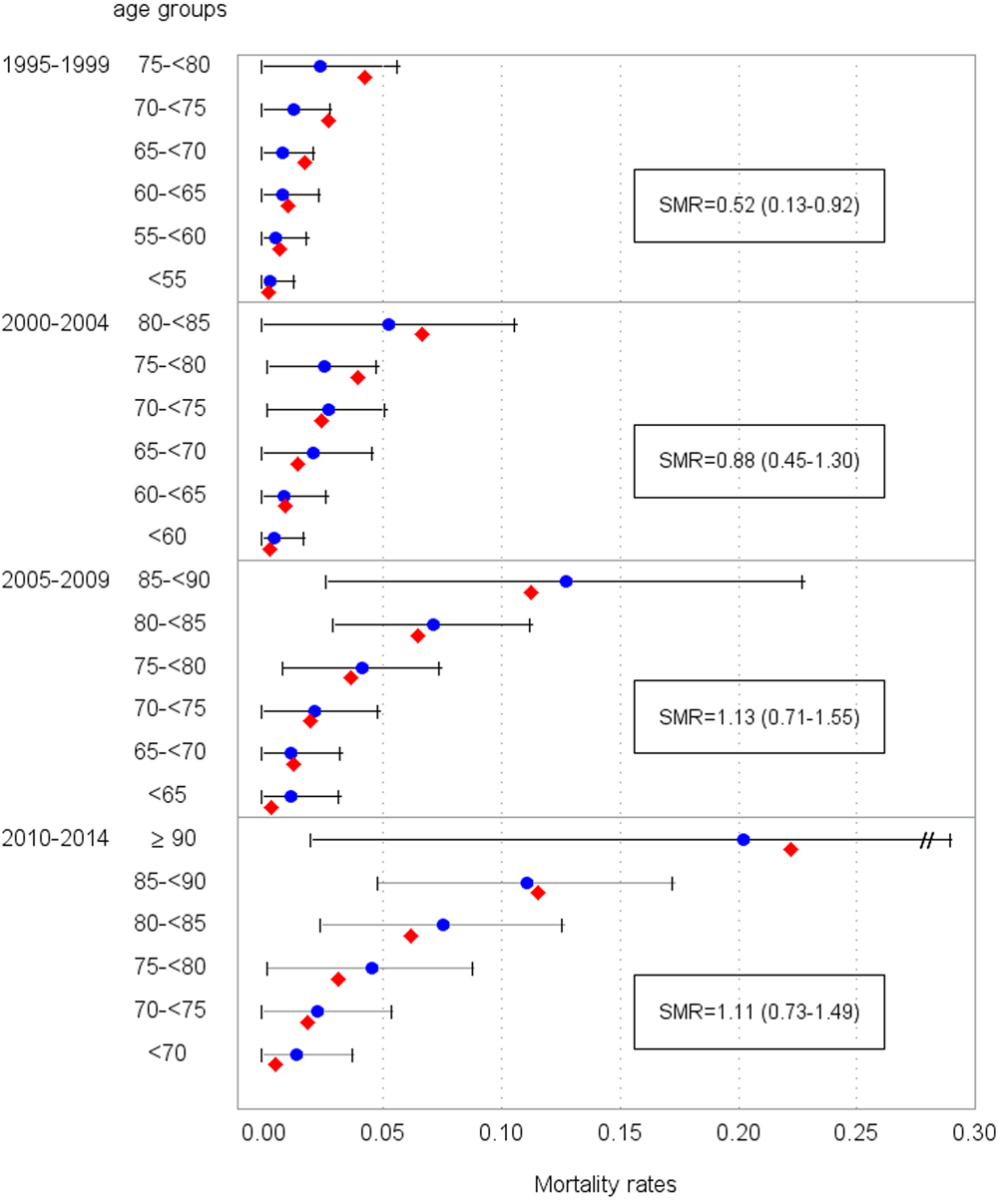
Age-specific mortality in 5-year periods after arthroplasty in the study cohort () (baseline recruitment Jan. 1995 until Dec. 1996) and general population (). SMR standardized mortality ratio.

As displayed in Fig. 2, a statistically significant decreased risk of mortality was observed in the first five years after hip or knee arthroplasty (1995–1999: overall SMR = 0.52; 95% CI 0.13-0.91) compared to general population. This association was more pronounced in the older age groups (>65 years). Mortality rates of the study cohort and the general population aligned in the subsequent five years interval. At the end of the observation period (2010–2014), mortality of the study cohort tentatively increased (SMR = 1.11; 95% CI 0.73-1.49), though the 95% CI was wide and included the null value.

### Associations with patterns of OA

Age was the primary risk factor in all three OA patterns, however, other risk factors were also identified (Supplementary Table S1). In our multivariate adjusted models higher age and self-reported history of overweight/obesity were hypothesized as risk factors for bilateral OA (aOR = 1.76; 95% CI 1.40-2.21 and aOR = 1.53; 95% CI 0.98-2.40, respectively). Higher age (aOR = 2.67; 95% CI 1.95-3.65) and higher cholesterol (aOR = 1.27; 95% CI 1.02-1.57) were associated with increased risk of generalized OA. Also age (aOR = 0.60; 95% CI 0.50-0.73) and cholesterol (aOR = 0.84; 95% CI 0.56-1.08) were associated with the cause of OA, indicating a risk decrease for secondary causes of OA in comparison to primary OA.

### Risk factors for all-cause mortality in patient groups with OA

As age, current smoking, and localization of OA were relevant factors for mortality in survival time analyses (Table 2), all further investigations of associations with other potential risk factors were adjusted for these variables in Model 1. Comorbidities associated with increased mortality were history of overweight/obesity, diabetes, gout, hypertension, and cardiac insufficiency. Among the laboratory findings, only high uric acid was statistically significantly associated with mortality. Bilaterality of OA was associated with higher mortality (aHR = 1.53; 95% CI 1.08-2.16) and patients with secondary OA had lower mortality than those with primary OA (aHR = 0.73; 95% CI 0.58-0.90). After mutual adjustment and variable selection (Model 2): age, current smoking, history of overweight/obesity, diabetes, hypertension, cardiac insufficiency, and uric acid revealed p-values below 0.10. Conversely, higher cholesterol was associated with a lower risk of mortality. Among the OA-patterns, secondary reasons of OA remained associated with lower mortality (aHR = 0.76; 95% CI 0.61-0.95), whereas a significant aHR of 1.53; 95% CI 0.95-2.45 was estimated for the category “unknown laterality of OA” indicating tentative association with higher mortality.

**Table 2:** Survival time analyses.

	Model 1^&^			Model 2*		
	aHR	95% CI	p-value	aHR	95% CI	p-value
Age (years; per SD)	**2.75**	**(2.36–3.20)**	<**0.0001**	**2.69**	**(2.29–3.16)**	<**0.0001**
Smoking (former vs. non-smoker)	0.96	(0.75–1.22)	0.72	0.95	(0.74–1.22)	0.70
Smoking (current vs. non-smoker)	**1.56**	**(1.12–2.17)**	**0.0083**	**1.48**	**(1.06–2.08)**	**0.021**
Localization (hip vs. knee)	**0.83**	**(0.68–1.02)**	**0.077**			
BMI (kg/m²; per SD)	1.06	(0.96–1.17)	0.25	**§**		
BMI (25–30 vs <25)	0.97	(0.74–1.26)	0.81	**§**		
BMI (30–35 vs. <25)	1.16	(0.87–1.54)	0.32	**§**		
BMI (>35 vs. <25)	1.22	(0.80–1.86)	0.36	**§**		
History of overweight/obesity (yes vs. no)	**1.37**	**(1.11–1.69)**	**0.0035**	**1.20**	**(0.97–1.82)**	**0.095**
Diabetes mellitus type 2 (yes vs. no)	**1.59**	**(1.18–2.15)**	**0.0024**	**1.33**	**(0.98–1.90)**	**0.067**
Gout (yes vs. no)	**1.37**	**(1.05–1.80)**	**0.023**			
Hypertension (yes vs. no)	**1.50**	**(1.22–1.84)**	**0.0001**	**1.31**	**(1.06–1.62)**	**0.014**
Cardiac infarction (yes vs. no)	1.40	(0.92–2.12)	0.11			
Cardiac insufficiency (yes vs. no)	**1.56**	**(1.24–1.96)**	**0.0002**	**1.32**	**(1.04–1.69)**	**0.014**
Hypercholesterolemia (reference: no)						
Hypercholesterolemia yes	0.91	(0.73–1.13)	0.38	**§**		
Unknown	1.00	(0.75–1.33)	0.99	**§**		
Cholesterol (mmol/l; per SD)	0.91	(0.81–1.02)	0.10	**0.88**	**(0.79–0.99)**	**0.035**
Uric acid (mmol/l; per SD)	**1.15**	**(1.03–1.29)**	**0.017**	**1.11**	**(0.99–1.24)**	**0.081**
hs-CRP (log mg/l; per SD)	1.08	(0.97–1.19)	0.16			
Laterality of OA (reference: unilateral)						
Bilateral OA	**1.53**	**(1.08–2.16)**	**0.017**	1.28	(0.89–1.82)	0.18
Unknown	**1.76**	**(1.12–2.76)**	**0.014**	**1.53**	**(0.95–2.45)**	**0.082**
Generalization of OA (reference: not generalized)						
Generalized OA	1.15	(0.91–1.46)	0.24			
Unknown	1.17	(0.91–1.52)	0.22			
Secondary OA (reference: primary)						
Secondary OA	**0.73**	**(0.58–0.90)**	**0.0040**	**0.76**	**(0.61–0.95)**	**0.017**
Unknown	**1.70**	**(1.01–2.88)**	**0.047**	1.36	(0.77–2.41)	0.29

aHR adjusted Hazard Ratios; CI confidence intervals; SD standard deviation for age 8.75 years, for BMI 4.28 kg/m^2^, for cholesterol 1.09 mmol/l, for uric acid 84.24 mmol/l, for CRP 1.06 log mg/l; OA osteoarthritis All models are statistically controlled for sex. Bold marked associations revealed p-values below 0.10. ^&^Model 1 shows hazard ratios with 95% confidence intervals adjusted for age, smoking status, and localization. *Model 2 shows all significant mutually adjusted hazard ratios with 95% confidence intervals after backward selection performed “by hand”. ^**§**^Variables not included in model 2 due to high overlap with other variables. In the final model, only variables with p-values less than 0.10 in at least one substratum were kept.

**Figure 3:**
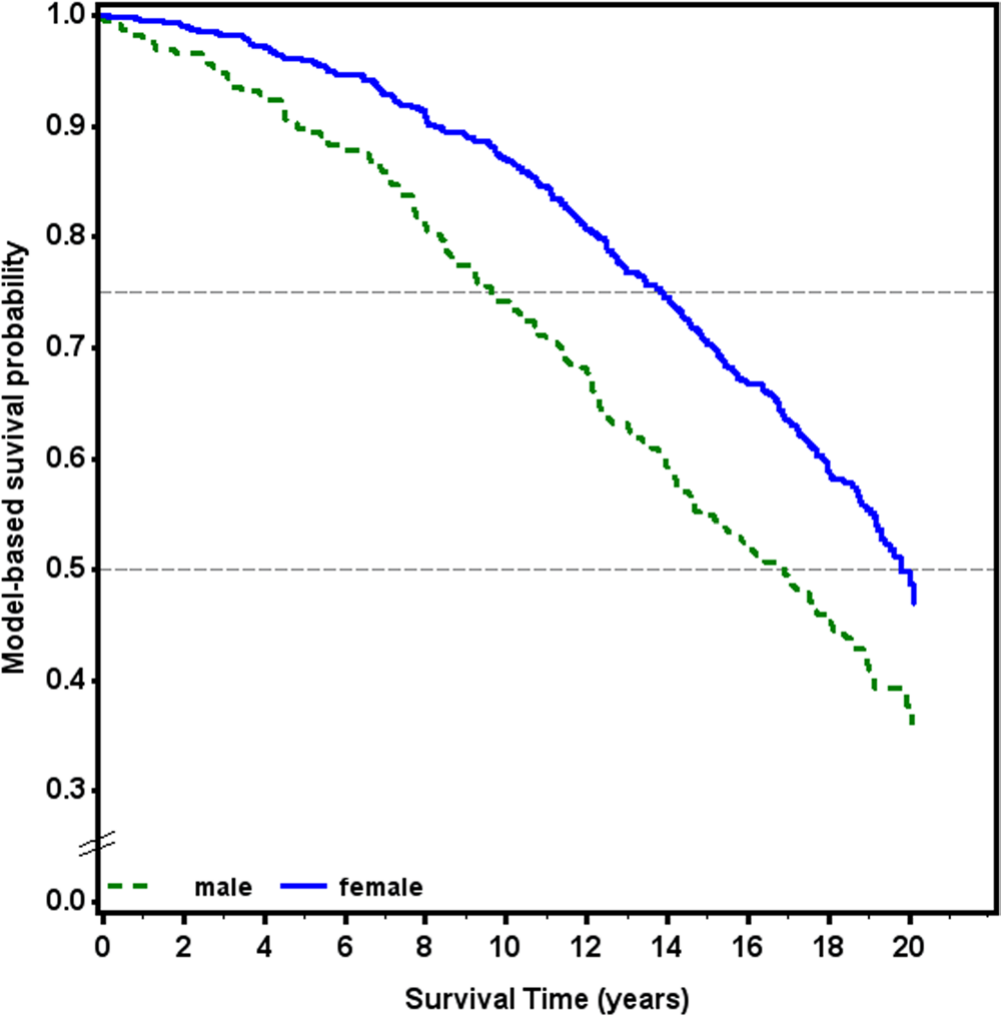
Adjusted survival probabilities stratified for sex. Estimated in Model 2 and adjusted for age, current smoking, self-reported history of overweight/obesity, diabetes, hypertension, cardiac insufficiency, uric acid, cholesterol, and laterality and secondary or primary cause of OA.

Figure 3 shows the survival probability differentiating between male and female patients. According to the adjusted survival probabilities estimated in Model 2, 25% of all male patients were deceased after about 9.5 years and 50% of all male patients were deceased after about 17 years. Among female patients, 25% were deceased after approximately 14 years and 50% after approximately 20 years.

## Discussion

The long-term follow-up of this large clinical cohort including patients who initially received a first arthroplasty of the hip or knee showed no statistically significant increased all-cause mortality compared to the general population. Though likely due to the selection for surgery, we even observed a lower mortality in the first 5 years. Furthermore, several cardiovascular risk factors such as hypertension and serum uric acid concentrations were associated with a higher risk of death during the follow-up period. Patients with secondary OA had a much lower risk of death compared to those with primary OA.

### Mortality of patients with OA compared to general population

Previous studies revealed inconsistent results concerning all-cause mortality in OA patients^10^. In 2008 Hochberg *et al*. described in a synthesis of 6 studies moderate evidence for elevated mortality in OA patients, particularly patients with knee OA or generalized OA^11^. Nuesch *et al*. also reported increased all-cause-mortality in a population-based English cohort with symptomatic hip and knee OA^12^. The major risk factors in the latter study included a history of diabetes, cancer, cardiovascular disease and walking disability. More recently, Barbour *et al*. found that radiographic hip osteoarthritis was associated with increased all-cause and cardiovascular disease mortality in older white women^13^.

In agreement with two previous studies on patients with knee joint replacement we found a reduced post-operative mortality in the first five years^14,15^. This observation could possibly be explained by a selection of relatively healthy patients for the surgical procedure compared to the general population. This suggestion is supported by the fact that the patterns were especially evident among more elderly patients. Furthermore, joint replacement usually improves physical activity of the patients and may thus also be a protective factor for cardiovascular fitness^16^ which represents an accepted predictor for all-cause mortality and cardiovascular events^17^. Interestingly, disability but not OA has recently been claimed to predict cardiovascular disease in a population-based cohort^18^. Possibly, the increased mortality in patients with bilateral OA may be explained by its effect on physical activity which may remain more impaired compared to patients with unilateral OA.

In contrast, reduced risk of mortality associated with the presence of OA compared to other chronic diseases has also been previously described^19^. Furthermore, no relevant association of knee or hip OA with all-cause mortality was found by Turkiewicz *et al*.^20^. A recent meta-analysis of currently available prospective cohorts throughout the world which investigate the association of symptomatic or radiological OA and all-cause mortality came to the conclusion that so far no reliable evidence exists for an association of OA and all-cause mortality^8^ and that more high-quality studies are required. These heterogeneous results indicate that the association between OA and mortality is quite complex and in addition to study quality, may be influenced by other factors such as selection of patients to surgical procedures, medical care seeking behavior, and disease phenotype and/or comorbidity issues^10^.

Focusing on OA patients who received the first arthroplasty for hip or knee OA as a reliable criterion for both symptomatic and radiologic OA, we also found no increased mortality compared to the general population after 20 years. However, the combination of reduced post-operative mortality within the first five years observed in our cohort and comparable mortality after 20 years points to an overall steeper increase in mortality. In addition, we found that various factors differentially influence OA patterns. Using this information, potential misclassifications especially in early stages of the disease which may be present in studies relying on health-care based databases could be excluded.

In addition to the influence of time after surgical therapy, an effect of pain and radiological OA pattern has been reported. Knee pain with or without radiological OA was associated with an increased mortality but no such association was seen for hand OA^21^. In another study symptomatic but not only radiographic hand OA was associated with an increased risk of coronary heart disease events^18,22^ In the current study, the definition of generalized OA included the presence of radiographic multi-site hand OA and was not associated with increased mortality. However, differentiation of symptomatic and asymptomatic hand OA was not performed.

### Risk factors for knee and hip OA

It has been suggested that metabolic syndrome and osteoarthritis share some common links^23–25^ but recently it has been reported that the associations might be lost after adjustment for BMI^26^. In the Ulm Osteoarthritis Study, several positive associations with laterality or generalization of OA were separately reported for baseline diabetes (risk factor for bilateral OA)^6^, BMI (risk factor for bilateral OA in patients with knee arthroplasty)^2^, cholesterol (risk factor for generalized OA in patients with knee arthroplasty)^3^, and uric acid (risk factor for generalized OA in patients with hip arthroplasty)^5^. In contrast no associations were found for hs-CRP^4^. The protective association of smoking with generalized OA has been reported in several studies. However, there was no consistent effect across studies^27^. Another recent study found even a higher protective association between smoking and presence of erosive hand OA in cross-sectional analyses than in the presented results but an increased risk in longitudinal analyses^22^. Their conclusion was that the contrasting results for smoking and hand OA suggested a lack of association. Together, these data now allow a comprehensive analysis of an association of these factors with long-term mortality.

### Risk factors for all-cause mortality in patient groups with OA

Besides age and current smoking, the localization/pattern of OA was also observed as a risk factor for all-cause mortality in the present study. As mentioned above, the increased risk in patients with bilateral OA may be associated with reduced physical activity and associated cardiovascular disease. In a similar manner, the history of overweight/obesity, diabetes, hypertension, and cardiac insufficiency were all associated with higher mortality in this cohort. As in another study, serum cholesterol was associated with a lower risk of mortality especially in subjects from the age of 50 years onward^28^. Furthermore, serum uric acid and gout were associated with increased mortality. Uric acid has been described to be associated with mortality in other contexts^29–31^. Interestingly, secondary OA was associated with significantly lower mortality compared to primary OA which suggests an involvement of systemic co-factors associated with primary OA in the determination of all-cause mortality. Because the subgroup of patients with secondary OA is rather small a more detailed analysis of its association with other metabolic or cardiovascular disease associated parameters was not possible. Moreover, this subgroup is quite heterogeneous with hip dysplasia in patients with osteoarthritis of the hip and joint trauma in patients with osteoarthritis of the knee representing the dominating underlying causes^32^.

### Strengths and limitations

The current study population with more than 800 patients was very well characterized at baseline and allows in most of the cases the definition of distinct subgroups. Detailed baseline examinations and assessment of laboratory data were also available. In addition, we had long term follow-up with very good response. Nevertheless, sample size limited our analyses of certain subgroups. Furthermore, due to German data protection rules we were not able to assess the specific causes of death including cardiovascular mortality. However, we would expect that associations with cardiovascular mortality would be even stronger than our current presented associations with all-cause mortality.

### Conclusions

Despite these limitations we saw no increased mortality in patients after first hip or knee arthroplasty after 20 years compared to the general population. However, a steeper increase in mortality over 20 years could be assumed. Cardio-metabolic risk factors were associated with an increased risk of bilateral OA and with lower long-term survival. Therefore, cardio-metabolic risk factors in patients at risk for OA and especially after arthroplasty should be consequently targeted to improve their overall survival.

## Methods

### Study population

Between January 1995 and December 1996 consecutive patients undergoing unilateral total hip or knee arthroplasty due to advanced OA were recruited in four hospitals in the South-West of Germany. In total, N = 809 patients fulfilled the inclusion criteria (i.e. white, age not exceeding 75 years, absence of malignancies, inflammatory diseases, or corticosteroid medication; no previous contralateral joint replacement) and gave written informed consent. The study and the current follow-up were approved by the Ethics Committee of Ulm University (No. 164/14) and were conducted according to the relevant guidelines and regulations. The baseline recruitment of the study has been described in detail elsewhere^32,33^.

### Study data collection and classification

In addition to demographic data (sex, age, smoking, weight, height), detailed information about history of physician diagnosed comorbidities and symptoms (e.g. diabetes mellitus type 2, gout, hypertension, cardiac infarction and insufficiency, overweight/obesity), drug use, and medical history regarding the ipsilateral and contralateral joint was gathered by standardized interview. Radiographic assessment of ipsilateral and contralateral joints and both hands was performed. The grading of degenerative changes in all joints was performed according to Kellgren and Lawrence criteria^34^. Kellgren severity grades ≥2 were defined as OA.

Patterns of OA were classified for generalization, laterality, and cause (primary or secondary)^33^. Briefly, OA was defined as “generalized” if OA was found in at least two finger joints and at least one first carpometacarpal joint in addition to the replaced knee or hip joint. Without involvement of OA of the hands, the disease pattern was classified as “not generalized”. Patients were classified as “unilateral” or “bilateral” based on absence or presence of OA in the contralateral joint. Potential risk factors of OA were assessed by self-reported medical history as well as radiographic evaluation. “Secondary OA” was assumed for the following reasons: infection, avascular necrosis and osteochondritis, hemorrhagic diathesis, traumatic events with radiologically and/or surgically confirmed structural joint lesions as well as sequelae of slipped femoral capital epiphysis and acetabular dysplasia in pelvic radiographs. If no evidence of these risk factors was present, an idiopathic origin of “primary OA” was assumed. Missing values in OA patterns occurred due to missing or incomplete radiographic or questionnaire assessment and were separately categorized as “unknown”.

Serum cholesterol levels and serum uric acid levels were obtained in non-fasting serum-samples samples taken preoperatively by standard venipuncture^3,5^. Hypercholesterolemia was defined as serum cholesterol level ≥6.2 mmol/l or the use of antihyperlipidemic drugs. Again, missing values were categorized as “unknown”. High sensitivity (hs-)CRP was measured in frozen serum samples after storing at −80 °C^4^ and log-transformed when used in regression analyses.

### Mortality

Mortality was assessed during the follow-up at 6, 12, and 60 months after the joint replacement and again after 18 to 20 years. Contact to the respective residents’ registration office was made in the year 2014 and if deceased, the exact date of death was obtained. Subjects still alive were contacted directly and further notifications of death were received in 2015. Last information was gathered on 31^st^ of August 2015. To compare the mortality of the cohort with the mortality of the general population in the South of Germany, data from the Federal Statistical Office were requested regarding population size and mortality of the population of Baden-Württemberg and Bayern from 1995 until the end of 2014^35^. Age groups for the comparison of the cohort and the general population were defined as follows: 25–54, 55–59, 60–64, 65–69, 70–74, 75–79, 80–84, 85–89, and ≥90 years.

### Statistical analysis

We compared age specific mortality rates with 95% confidence intervals (CI) between the cohort and the general population. We calculated standardized mortality ratios (SMR) with 95% confidence intervals in 5 year-steps for 1995–1999, 2000–2004, 2005–2009, and 2010–2014. Because of missing values for laboratory findings (n = 126 for cholesterol, n = 110 for uric acid, and n = 39 for hs-CRP) and single missing values in the comorbidities (each n = 1 for diabetes, cardiac infarction, and cardiac insufficiency, n = 2 for gout), we performed multiple imputation according to Rubin’s rule^36^ before applying regression models. Estimation was based on Markov Chain Monte Carlo (MCMC) with ten imputations. We estimated adjusted odds ratios (aOR) with 95% confidence intervals to describe the association of baseline covariates with the various patterns of OA using logistic regression models. In Model A all associations were adjusted for age. In the multivariate Models B completely and mutually adjusted aORs were determined for all covariates.

In addition, we estimated adjusted hazard ratios (aHR) with 95% confidence intervals (CI) using proportional hazards models to assess the association of the various OA patterns and baseline covariates with all-cause mortality. The proportional hazards assumption was tested by an interaction term between log(time) and the covariates. To achieve fulfillment of the proportional hazard assumption the variable sex was included in a STRATA-statement and therefore, no effect estimates for this variable were available. In Model 1 hazard ratios were adjusted for age, smoking status, and localization of OA. Model 2 shows mutually adjusted hazard ratios after investigator driven backward selection. In the final model, only variables with p-values less than 0.10 in at least one substratum were kept. Model 2-based adjusted survival probabilities were retrieved stratified for men and women and displayed graphically in survival curves. P-values from statistical testing were used for explorative data analyses only. All analyses were performed using SAS 9.4 (SAS Institute Inc., Cary, NC, USA).

### Data availability statement

Due to ethical restrictions, the data cannot be made publicly available but are available upon request. The request should be directed to Prof. Rothenbacher (dietrich.rothenbacher@uni-ulm.de).

## Electronic supplementary material

Supplementary Table S1
