# Supplementary material for: Osteoarthritis-patterns, cardio-metabolic risk factors and risk of all-cause mortality: 20 years follow-up in patients after hip or knee replacement
Source: Sci Rep. 2018 Mar 27;8:5253. doi: 10.1038/s41598-018-23573-2 (PMC5869736; doi:10.1038/s41598-018-23573-2)
Supplement: Supplementary file 1 — Supplementary Table S1 [file 41598_2018_23573_MOESM1_ESM.pdf]

Osteoarthritis-patterns, cardio-metabolic risk factors and risk of all-cause mortality: 20 years follow-up in patients after hip or knee replacement.

Büchele G[1], Günther KP[2], Brenner H[3,4], Puhl W[5], Stürmer T[6], Rothenbacher D[1,*], Brenner RE[7]

[1] Institute of Epidemiology and Medical Biometry, Ulm University, Helmholtzstraße 22, 89081 Ulm, Germany

[2] University Center of Orthopaedics and Traumatology, University Medicine Carl Gustav Carus Dresden, TU Dresden, Dresden, Germany

[3] Division of Clinical Epidemiology & Aging Research, German Cancer Research Center (DKFZ), Heidelberg, Germany

[4] Network Aging Research, University of Heidelberg, Germany

[5] Department of Orthopedics(emeritus), University of Ulm, Ulm 89081, Germany

[6] Department of Epidemiology, Gillings School of Global Public Health, University of North Carolina at Chapel Hill, Chapel Hill, NC, USA.

[7] Department of Orthopedics, Division for Biochemistry of Joint and Connective Tissue Diseases, University of Ulm, Ulm 89081, Germany.

*Correspondence to:

Prof. Dr. med. Dietrich Rothenbacher, MPH

Institute of Epidemiology and Medical Biometry, Ulm University, 89081 Ulm Germany

dietrich.rothenbacher@uni-ulm.de

Supplementary Table S1: Associations with pattern of osteoarthritis (OA)

	Bilateral OA (vs. unilateral)			Generalized OA (vs. not generalized)			Secondary OA (vs. primary)		
	**aOR**	**95% CI**	**p-value**	**aOR**	**95% CI**	**p-value**	**aOR**	**95% CI**	**p-value**
**Model A[&]**									
Age (years; per SD)	*1.84*	*(1.53-2.21)*	*<.0001*	*3.07*	*(2.32-4.07)*	*<.0001*	*0.58*	*(0.49-0.67)*	*<.0001*
Sex (female vs. male)	1.01	(0.66-1.55)	0.96	*1.91*	*(1.25-2.92)*	*0.0029*	0.84	(0.62-1.15)	0.28
Smoking (former vs. non-smoker)	0.96	(0.60-1.55)	0.88	*0.53*	*(0.34-0.82)*	*0.0045*	1.24	(0.89-1.74)	0.21
Smoking (current vs. non-smoker)	1.11	(0.60-2.06)	0.73	*0.53*	*(0.25-1.13)*	*0.099*	1.12	(0.70-1.80)	0.64
Localization (hip vs. knee)	1.04	(0.66-1.64)	0.86	*0.71*	*(0.48-1.04)*	*0.077*	0.98	(0.72-1.35)	0.92
BMI (kg/m²; per SD)	1.17	(0.94-1.46)	0.16	1.08	(0.89-1.30)	0.44	*0.86*	*(0.73-1.00)*	*0.051*
BMI (25-30 vs <25)	1.11	(0.67-1.82)	0.69	1.16	(0.70-1.93)	0.56	0.77	(0.53-1.13)	0.18
BMI (30-35 vs. <25)	1.51	(0.81-2.80)	0.19	0.99	(0.57-1.73)	0.99	0.81	(0.52-1.25)	0.34
BMI (>35 vs. <25)	2.69	(0.77-9.35)	0.12	1.49	(0.68-3.27)	0.32	*0.50*	*(0.24-1.02)*	*0.055*
History of overweight/obesity (yes vs. no)	*1.56*	*(1.02-2.37)*	*0.038*	1.34	(0.91-1.98)	0.14	*0.73*	*(0.54-0.99)*	*0.042*
Hypercholesterolemia (yes vs. no)	0.91	(0.57-1.46)	0.70	*1.65*	*(1.10-2.47)*	*0.016*	0.78	(0.55-1.10)	0.15

Cholesterol (mmol/l; per SD)	0.87	(0.70-1.09)	0.21	*1.33*	*(1.08-1.64)*	*0.0066*	*0.83*	*(0.69-1.01)*	*0.065*
Uric acid (mmol/l; per SD)	0.91	(0.73-1.14)	0.41	1.18	(0.96-1.46)	0.12	0.97	(0.82-1.15)	0.73
hs-CRP (log mg/l; per SD)	1.03	(0.84-1.27)	0.77	1.05	(0.86-1.29)	0.60	0.99	(0.84-1.15)	0.86
Diabetes mellitus type 2 (yes vs. no)	2.32	(0.81-6.65)	0.12	1.11	(0.60-2.06)	0.74	0.63	(0.36-1.12)	0.12
Gout (yes vs. no)	1.60	(0.78-3.29)	0.20	*1.71*	*(1.01-2.89)*	*0.045*	0.76	(0.48-1.20)	0.24
Hypertension (yes vs. no)	*1.45*	*(0.93-2.25)*	*0.098*	*1.48*	*(1.00-2.18)*	*0.047*	0.84	(0.61-1.14)	0.25
Cardiac infarction (yes vs. no)	3.91	(0.52-29.2)	0.18	0.77	(0.32-1.83)	0.55	0.55	(0.23-1.28)	0.17
Cardiac insufficiency (yes vs. no)	1.27	(0.66-2.52)	0.50	*1.67*	*(1.08-2.60)*	*0.023*	0.79	(0.52-1.22)	0.29
**Model B***									
Age (years; per SD)	**1.76**	**(1.40-2.21)**	**<.0001**	**2.67**	**(1.95-3.65)**	**<.0001**	**0.60**	**(0.50-0.73)**	**<.0001**
Sex (female vs. male)	0.99	(0.59-1.67)	0.98	1.49	(0.88-2.52)	0.14	0.89	(0.61-1.31)	0.56
Smoking (former vs. non-smoker)	1.00	(0.59-1.70)	0.99	**0.60**	**(0.36-0.99)**	**0.047**	1.23	(0.84-1.79)	0.29
Smoking (current vs. non-smoker)	1.18	(0.61-2.29)	0.61	0.66	(0.30-1.46)	0.30	1.08	(0.65-1.80)	0.76
Localization (hip vs. knee)	1.16	(0.72-1.88)	0.53	0.87	(0.57-1.32)	0.50	0.86	(0.61-1.21)	0.38

History of overweight/obesity (yes vs. no)	**1.53**	**(0.98-2.40)**	**0.062**	1.02	(0.66-1.57)	0.93	0.78	(0.56-1.08)	0.13
Cholesterol (mmol/l; per SD)	0.87	(0.69-1.09)	0.23	**1.27+**	**(1.02-1.57)**	**0.029**	**0.84**	**(0.56-1.08)**	**0.087**
Uric acid (mmol/l; per SD)	0.84	(0.64-1.09)	0.18	1.19	(0.94-1.52)	0.15	1.00	(0.82-1.22)	0.98
hs-CRP (log mg/l; per SD)	0.98	(0.79-1.22)	0.85	1.03	(0.83-1.28)	0.81	1.03	(0.87-1.21)	0.76
Diabetes mellitus type 2 (yes vs. no)	2.01	(0.69-5.90)	0.20	0.97	(0.49-1.91)	0.93	0.64	(0.35-1.15)	0.14
Gout (yes vs. no)	1.75	(0.81-3.78)	0.15	1.47	(0.82-2.65)	0.20	0.78	(0.48-1.28)	0.34
Hypertension (yes vs. no)	1.39	(0.87-2.22)	0.17	1.24	(0.81-1.89)	0.32	0.96	(0.69-1.33)	0.79
Cardiac infarction (yes vs. no)	3.33	(0.44-25.3)	0.25	0.83	(0.33-2.10)	0.70	0.54	(0.23-1.31)	0.17
Cardiac insufficiency (yes vs. no)	1.05	(0.52-2.16)	0.89	1.38	(0.85-2.24)	0.20	0.93	(0.59-1.46)	0.74

aOR adjusted Odds Ratios; CI confidence intervals; SD standard deviation for age 8.75 years, for BMI 4.28 kg/m^2, for cholesterol 1.09 mmol/l, for uric acid 84.24 mmol/l, for CRP 1.06 log mg/l

& Model A shows age adjusted odds ratios with 95% confidence intervals. Italic marked associations revealed p-values below 0.10.

*** Model B** shows completely and mutually adjusted odds ratios with 95% confidence intervals. Not included in Model B were variables regarding BMI and hypercholesterolemia due to high overlap with other variables. Bold marked associations revealed p-values below 0.10.
